# Measuring biventricular function and left atrial volume in a single five-dimensional whole-heart cardiovascular magnetic resonance scan at 0.55T

**DOI:** 10.1016/j.jocmr.2025.101906

**Published:** 2025-05-08

**Authors:** Xavier Sieber, Katherine Binzel, Juliet Varghese, Yingmin Liu, Jerome Yerly, Christopher W. Roy, Panagiotis Antiochos, Milan Prsa, Ruud B. van Heeswijk, Orlando P. Simonetti, Matthias Stuber

**Affiliations:** a Department of Diagnostic and Interventional Radiology, Lausanne University Hospital (CHUV) and University of Lausanne (UNIL), Lausanne, Switzerland; b Dorothy M. Davis Heart and Lung Research Institute, The Ohio State University, Columbus, Ohio, USA; c Department of Biomedical Engineering, The Ohio State University, Columbus, Ohio, USA; d CIBM Center for Biomedical Imaging, Lausanne, Switzerland; e Department of Cardiology, Lausanne University Hospital (CHUV), Lausanne, Switzerland; f Woman-Mother-Child Department, Lausanne University Hospital and University of Lausanne, Lausanne, Switzerland; g Department of Radiology, The Ohio State University, Columbus, Ohio, USA; h Department of Internal Medicine, Division of Cardiovascular Medicine, The Ohio State University, Columbus, Ohio, USA

**Keywords:** 5D, Low-field, 0.55T, Biventricular function, Free-running, Self-gated

## Abstract

**Background:**

Cardiovascular magnetic resonance (CMR) has not seen widespread adoption beyond large urban academic centers. The reasons for this limited uptake include the cost, time-intensive nature, and special expertise of CMR. Self-navigated five-dimensional (5D), x-y-z-cardiac-respiratory, free-funning whole-heart CMR using self-navigation (5D CMR) implemented on a low-field clinical scanner may help bridge this gap for biventricular function assessment and left atrium volume index measurement.

**Methods:**

Whole-heart three-dimensional radial phyllotaxis balanced steady-state free precession data were collected in ten healthy adult subjects. Self-navigation was used to extract respiratory and cardiac motion signals and to generate motion-resolved 5D CMR datasets. The right- and left-ventricular ejection fraction (RVEF and LVEF), left atrial volume index, contrast ratio, sharpness, perceived image quality, and total scan durations were ascertained and compared to those obtained with the reference 2D cine images.

**Results:**

5D CMR allowed for time-efficient and concordant measurements when compared to the 2D reference method. The 5D CMR images resulted in lower CR on 5D CMR images (3.3 ± 2.9) than on 2D cines (4.7 ± 1.2), and similarly lower perceived image quality (1.8 ± 0.8 for 5D CMR and 3.6 ± 0.9 for the 2D cines). However, the LVEF measurements were similar with no statistically significant differences (Mean: 58 ± 5% for 5D CMR and 59 ± 5% for 2D cine, p = 0.49) and the LoA were low (–2.81% to 3.81%). For the RVEF, the measurements were also in good agreement when RVEF was measured on the axial views (60 ± 3% for 5D CMR and 60 ± 4% for 2D cine, p = 0.85) and the LoA were also low (–2.87% to 3.07%).

**Conclusion:**

5D CMR without the need for electrocardiogram, breath-holding, navigators, or complex scan planning enables a highly simplified and time-efficient assessment of biventricular cardiac function on a 0.55T clinical system in 7:50 min.

## Graphical abstract

**Figure:**
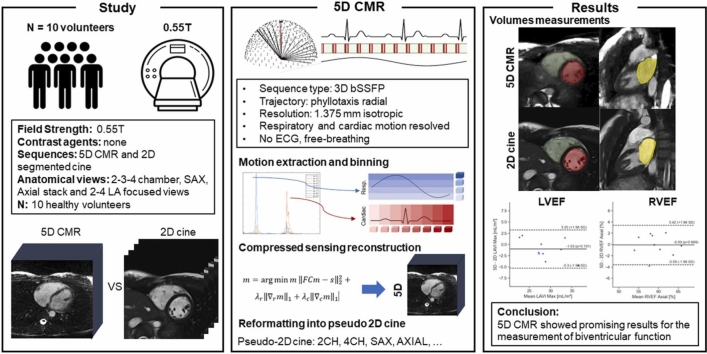

## 1. Introduction

Cardiovascular disease results in significant morbidity and mortality, making it the leading cause of death in industrialized nations and posing a substantial burden on global healthcare systems [1]. In addressing this, non-invasive cardiac evaluation is essential, with cardiovascular magnetic resonance (CMR) being is accepted as the gold standard for the assessment of cardiac function, structure, and injured myocardium [2]. However, associated costs [3], [4], [5] and required expertise [6], [7] may be prohibitive for widespread adoption. Therefore, ongoing efforts for a broader adoption of CMR are directed at streamlined protocols that are faster and easier to use.

Routine CMR imaging requires the use of breath-holds, navigators, and electrocardiogram (ECG) to avoid motion artifacts or to resolve the motion of the heart [8], [9], [10], [11], [12]. Recently, five-dimensional (5D) free-running whole-heart CMR using total self-navigation (5D CMR) has been introduced [13]. In 5D CMR, three-dimensional (3D) radial data are collected continuously, uninterrupted, without the need for ECG lead placement and synchronization, breath-holding, or complex scan plane and respiratory navigator planning. As a result, data collection is highly efficient, ease-of-use is maximized, and image data can retrospectively be examined both in time and space. Therefore, 5D CMR may be a good candidate to overcome some of the abovementioned challenges.

Three-dimensional visualization of the entire heart, encompassing chambers, valves, vessels, and surrounding structures, is crucial for detecting complex cardiac conditions, congenital heart defects, and anatomical variations that may elude traditional 2D imaging. Additionally, acquiring spatially isotropic 3D volumes grants the flexibility to retrospectively create slice orientations that were not planned during the scan—including axial slices which are precise for RVEF quantification [14], and dedicated left atrium views which provide more accurate atrial volume measurements [15]. While such progress may ultimately help disseminate CMR outside of large, academic urban centers, a more global reach may even be expected if such 5D CMR were implemented on modern low-field MR equipment [16]. Contemporary low-field MR systems are marketed at a lower purchase price, maintenance and service costs are reduced, helium refills are no longer needed, and without quench lines and a lower magnet weight, siting constraints are much less stringent than for systems with higher field strength.

Low-field magnetic resonance imaging (MRI) has already shown promise for cardiovascular applications for several reasons. Firstly, the lower magnetic field strength is associated with reduced B0 inhomogeneity and magnetic field susceptibility, which can be particularly advantageous when imaging structures near air-filled cavities, such as the heart and blood vessels [17]. Together with favorable T1 and T2 relaxation times, this puts balanced steady-state free precession (bSSFP), the work horse of 1.5T CMR into focus, as banding artifacts are spaced further apart at low field, and as this in turn enables the reduction of the signal readout bandwidth in the interest of improved signal-to-noise ratio [17]. Overall, these factors make low-field MRI a promising option for cardiovascular applications, providing a balance between imaging capabilities, cost-effectiveness, and patient experience.

For these reasons, we have implemented, optimized, and tested a fully self-gated free-breathing 5D CMR bSSFP sequence on a 0.55T clinical system with moderate gradient performance. The first results obtained in healthy volunteers are reported. Ejection fraction measurements of the left and right ventricle as well as minimum and maximum volume indices of the left atrium were measured and compared to the values obtained from reference standard 2D cine acquisitions in the same subjects.

## 2. Methods

### 2.1. Pulse sequence optimization

We implemented and optimized a free-running 3D radial phyllotaxis bSSFP pulse sequence (Table 1) from which the 5D CMR images were reconstructed [18], [19]. During the optimization procedure, the radiofrequency (RF) excitation angle was adjusted experimentally, from 50° to 130 in steps of 20°, to determine the maximal blood-myocardium contrast ratio (CR) defined as:

(1) $$\mathrm{CR}=\frac{S_{b}}{S_{m}}$$

where $S_{b}$ and $S_{m}$ are the mean signal intensity in the left ventricular blood pool and in the myocardium respectively. ROIs were drawn manually on each image.

**Table 1:** Protocol parameters for the 2D cine and 5D CMR used in this study.

	2D cine	5D CMR
ECG gating	Retrospective	None
TE/TR (ms)	1.95/4.20	2.98/5.94
FOV (mm)	288 × 360	220 × 220 × 220
Acquisition matrix size	192 × 154	160 × 160 × 160
Image matrix size (px)	304 × 384	160 × 160 × 160
Acquired Spatial resolution(mm^2^)	2 × 2	1.4 × 1.4 × 1.4
Reconstructed resolution (mm^2^)	1 × 1	1.4 × 1.4 × 1.4
Slice thickness (mm)	8	8 (after reformatting)
RF excitation angle (°)	100	110
Bandwidth (Hz/px)	930	401
Number of cardiac frames	25	17–22
Number of breath-holds	25–35	0
Breath-hold duration [sec]	8	0
Breath-hold recovery [sec]	10	0
Number of slices	34 ± 2	160
Acquisition time [min:sec]	2:58 ± 31	7:50
Total exam time [min:sec]	60:26 ± 37:12	7:50
Reconstruction type	Compressed sensing	Compressed sensing
Undersampling factor	2.2	c.a. 50

The exam time is the sum of the acquisition time, patient recovery time, slice planning, repeated slice, electrode replacement, and ECG trigger filter recalibration

*2D* two-dimensional, *5D* five-dimensional, *CMR* cardiovascular magnetic resonance, *ECG* electrocardiogram, *TE* echo time, *TR* repetition time, *FOV* field-of-view, *RF* radiofrequency

*Data are means ± standard deviation.*

To allow experiments with a broader range of RF excitation angles, the RF pulse duration of the 5D CMR sequence was set to 1 ms. Next, the receiver bandwidth was optimized by visually comparing the banding artifacts induced by longer TRs as well as the SNR in the blood compartment of a phantom in static images, i.e. without compressed sensing, acquired with bandwidths of 801, 601, 401 and 201 Hz/px. The SNR was measured as

(2) $$\mathrm{SNR}=\frac{S_{b}}{\sigma_{n}\;}$$

Where $S_{b}$ is the average signal measured in a region-of-interest (ROI) manually drawn in the “blood” compartment of the phantom and $\sigma_{n}\;$is the standard deviation of the signal in an ROI outside the phantom.

The total number of lines was selected to ensure that each bin contains more than 5% of the Nyquist limit. We assumed 80 3D images, four respiratory states with 20 cardiac states, which corresponds to a heart rate of 60 bpm with a cardiac temporal resolution of 50 ms. Our acquisition matrix size was 160 × 160 × 160 points, $N_{p}=160$. Using the Nyquist limit for radial imaging formula, we computed the total number of lines as follows:

(3) $$N_{\lim}>\frac{\pi }{4}\cdot N_{p}^{2}\cdot N_{\mathrm{im}}\cdot U_{\mathrm{factor}}=80425\left[\mathrm{lines}\right]$$

Where $N_{\lim}$ is the number of lines needed to reach 5% of the Nyquist limit, $N_{\mathrm{im}}=80$ is the number of images and $U_{\mathrm{factor}}=5\%\;$is the undersampling factor.

As part of the free-running sequence, a superior-inferior (SI) readout was acquired every 18 k-space lines to extract the respiratory and cardiac motion signals. The number of k-space lines interleaved between each SI readout was selected to remain close to a SI sampling rate of one per 100 ms, which provides accurate cardiac signal extraction. Therefore, we chose 18 lines including the SI readout, which yielded one SI readout every 107 ms for a TR of 5.94 ms. With 18 readouts per phyllotaxis pattern, we get a minimal number of phyllotaxis patterns, $N_{\mathrm{shots}}$, of:

(4) $$N_{\mathrm{shots}}=\frac{N_{\lim}}{18}=4446$$

Since not every phyllotaxis rotation leads to a continuous and smooth sequence of readouts [19], which is required to minimize eddy currents effects, we selected the closest phyllotaxis number that satisfied that criterion, $N_{\mathrm{shots}}$ = 4529 and therefore $N_{\mathrm{readouts}}$= 81,522 radial readouts. This choice of parameters yielded an acquisition time (TA) of 7:50 [min:sec].

For reference standard comparison, breath-held ECG-gated segmented k-space bSSFP cine images (2D cine) were acquired at multiple anatomical levels [20]. This sequence was always acquired in end expiration to provide consistent EF measurement when comparing to those of the 5D CMR [21]. Data were collected during one breath-hold per slice, using a readout bandwidth of 930 Hz/px, and TE/TR of 1.95/4.2 ms (Table 1). The 2D cine images were acquired in short-axis (SAX) and axial stacks (AX) covering the heart, and long axis views including the two-chamber (2CH), four-chamber (4CH), as well as LA-focused 2CH and 4CH views (LA2CH and LA4CH, respectively). The 2D cine had an in-plane resolution of 2 × 2 mm^2^, a slice thickness of 8 mm and a 2 mm gap between slices. For each slice, 25 frames were acquired over 8 heartbeats. The data w reconstructed using compressed sensing with an acceleration factor of 2.8 (Table 1).

### 2.2. CMR acquisition

The human study was approved by the local Institutional Review Board and written informed consent was provided by all participants. Data were collected in 10 healthy adult subjects (6 female, age 29 ± 6 years, Table 2) on a 0.55T clinical scanner (MAGNETOM Free.Max, Siemens Healthineers AG, Erlangen, Germany). The bore diameter of the system is 80 cm, and the gradients have a maximum amplitude of 26 mT/m and slew rate of 45 T/m/s. A large flexible six-element phased array receiver coil (two rows of three elements each) in combination with the nine spine coil elements (three rows of three elements each) located under the patient table were used for signal reception. Free-running data were collected using the above sequence during free breathing with the heart centered in the acquired volume. External ECG trigger inputs used for the 2D cines were provided by MRI compatible patient monitoring devices (3880 MRI Patient Monitoring System, IRADIMED, Winter Springs, Florida or Expression MR400, Philips N.V., Amsterdam, The Netherlands).

**Table 2:** Characteristics of the healthy volunteer population.

Number of healthy volunteers	10
Age [y]	29 ± 6
Heart rate [bpm]	66 ± 10
Weight [kg]	74 ± 14
Height [cm]	169 ± 6
BMI [kg/m^2^]	26 ± 4
Reference LVEF (2D cine)	59 ± 4%
Reference RVEF (2D cine)	58 ± 4%
Sex	6 females/4 males

*BMI* body mass index, *LVEF* left-ventricular ejection fraction, *RVEF* right-ventricular ejection fraction

*Data are means ± standard deviation.*

### 2.3. Image reconstruction

**Fig. 1:**
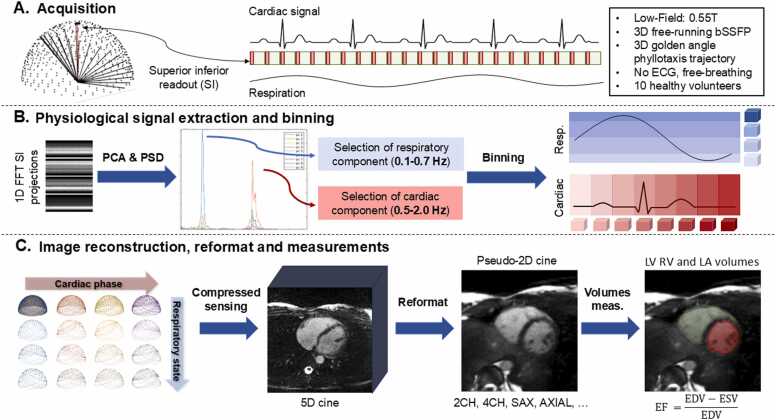
Schematic view of the 5D CMR acquisition, reconstruction, and processing. The pulse sequence consists of an interleaved 3D radial bSSFP sequence with a phyllotaxis pattern that starts each segment with a SI readout for self-navigation purposes. The SIs are extracted, and PCA is applied to its frequency spectrum to extract the cardiac and respiratory motion and to bin all readouts accordingly. Lastly, the 5D images are reconstructed and reformatted to obtain 2D cines with 8 mm slice thickness on which LV, RV, and LA volumes are measured at peak systole and end diastole. *5D* five-dimensional, *CMR* cardiovascular magnetic resonance, *3D* three-dimensional, *bSSFP* balanced steady-state free precession, *SI* superior-inferior, *PCA* principal component analysis, *LV* left ventricle, *RV* right ventricle, *LA* left atrial

The inverse Fourier transform of the SI readouts along each coil were combined in a single matrix and examined using principal component analysis (PCA) along its spatial dimension, to select the components that best represent cardiac and respiratory motion [13]. The physiological signals were interpolated to match the timing of each k-space line. The k-space data were then sub-divided into 50 ms cardiac and 4 equally populated respiratory bins. Each readout was sorted based on the position of the heart inferred from the SI readout. Compressed sensing with total variation (TV) regularization along the cardiac and respiratory dimension was used to reconstruct the 5D CMR images (Fig. 1) [13]. The reconstruction was performed by solving the following optimization problem:

(5) $$m=\arg\min m{\left\|\mathrm{FCm}-s\right\|}_{2}^{2}+\lambda_{r}{\left\|\nabla_{r}m\right\|}_{1}+\lambda_{c}{\left\|\nabla_{c}m\right\|}_{1}$$

where m is the resultant 5D reconstructed image, F is the nonuniform fast Fourier transform operator, C refers to the coil sensitivities, s is the acquired k‐space data set, ∇_c_ and ∇_r_ are the first‐order finite difference operators along the cardiac and respiratory dimensions, respectively, and λ_c_ and λ_r_ represent the corresponding regularization weights. The regularization weights, λ_c_ and λ_r_, were experimentally optimized to generate satisfactory image quality for segmentation without compressing the cardiac and respiratory motion. We reconstructed data for one volunteer using $\lambda_{c}=[0.001;0.0025;0.005;0.0075;0.01;0.025;0.05]$ and then, with the optimal λ_c_, we tested a range of $\lambda_{r}=[0.001;0.002;0.005;0.01;0.02;0.05;0.1]$. The optimal weight combination was ascertained by consensus reading of four coauthors (XS, RBvH, MS, and OPS). The optimization problem was solved using alternating direction method of multipliers (ADMM) and nonlinear conjugate gradient methods. The 5D CMR images were reconstructed offline on a Linux workstation with two 24-core CPUs (Intel Xeon Gold 6248 R, Intel, Santa Clara, California), 1.5 TB of RAM, and a Nvidia RTX A6000 GPU (Nvidia, Santa Clara, California) using Matlab R2021b (The MathWorks, Natick, Massachusetts).

### 2.4. Image reformatting and analysis

The 5D CMR images were reformatted to correspond with the 2D cine slice orientations. The end expiration frame in 5D CMR scans was selected for quantitative analysis as it has the sharpest blood-myocardium boundary and had lower levels of streaking artifact. A reformatted slice thickness of 8 mm was used to match the 2D cine slice thickness.

To assess the image quality quantitatively, two metrics were computed on the resulting 5D images and the 2D cine in AX, SAX, LA2CH and LA4CH in both end diastolic and peak systolic phases. The first metric used was the CR between left ventricular blood pool and in the myocardium. ROIs were drawn manually on each image. This metric aims at quantifying the expected loss of blood-myocardium contrast due to the 3D nature of the proposed sequence. The variance of the Laplacian (LAPV) was measured by taking the variance over a ROI centered on the heart filtered by a 3 × 3 Laplacian filter which highlights the edges of the image. This metric quantifies the variance of the edges in the ROI which is an indicator of sharpness [22].

Additionally, two cardiologists with 10 years and 12 of experience reviewed the visual quality of the cines and graded the images according to a Likert scale between 1 and 5 (1, non-diagnostic; 2, poor; 3, acceptable; 4, good; 5, excellent image quality). One mid ventricular slice for the SAX, one basal slice for the AX, the LA2CH slice and the LA4CH slice were selected for all volunteers for image scoring. A linear fit was applied to the CR, LAPV, and Likert scores against participant BMI and RR interval duration to assess their influence on image quality.

The left ventricular (LV) and right ventricular (RV) ejection fraction (EF) were measured with CVI42 (Circle CVI, Calgary, Alberta, Canada) for the 5D CMR and SuiteHeart (Neosoft, LLC, Pewaukee, Wisconsin) for the 2D cine by two different observers. Endocardial contours were automatically segmented by the software and manually adjusted by the observers on the end systolic (ES) and end diastolic frames (ED). The LVEF was measured on the routinely acquired SAX images using 2CH and 4CH views to track the valve. The RVEF was measured both on the SAX images and AX images to compare the accuracy and utility of the two views [23].

Left atrium minimum (LAVI_min_) and maximum (LAVI_max_) volume indices were measured on the LA-focused 2CH and 4CH views using the biplane area-length algorithm [15]:

(6) $$\mathrm{LAVI}=\;\frac{8\;A_{1}{\;A}_{2}}{3\pi L_{\min}\mathrm{BSA}}$$

where $A_{1}$ and $A_{2}$ are the area of the left atrium blood pool measured on the LA2CH and LA4CH views, $L_{\min}$ is the shortest long-axis length measured in either view and BSA is the body surface area computed with the Du Bois formula [24].

Contrast ratio, LAPV and Likert score were compared in a box plot. Additionally, linear correlations were estimated between the two methods and the R-squared value, R^2^, was reported for each measurement. Bland–Altman analysis was performed on the LV and RV ejection fraction measurements and for the LA volumes measurements. Based on the Bland–Altman analysis, 95% Limits of Agreements (LoA) and bias were reported. Statistical significance was calculated using a paired Wilcoxon signed-rank test with p<0.05 considered significant.

## 3. Results

### 3.1. Pulse sequence optimization

The RF excitation angle was limited to 85° when using an RF pulse of 600 us, therefore the duration of the RF excitation was extended to 1 ms to reach an RF excitation angle (FA) of up to 130°.

**Fig. 2:**
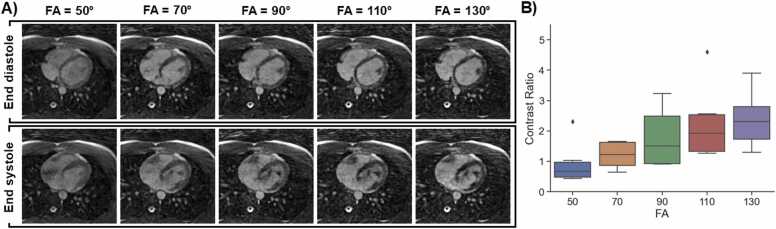
A) Axial free-running images with RF excitation angles ranging from 70° to 130° in steps of 20°, B) Boxplot of CR between blood and myocardium measured at end diastole and end systole as a function of the RF excitation angle. The CR steadily increases with the RF excitation angle in the examined range. *RF* radiofrequency, *CR* contrast ratio

The CR in the images with different FAs was 0.93±0.71, 1.21±0.45, 1.78±1.01, 2.26±1.28 and 2.39±0.95 for 50°, 70°, 90°, 110° and 130°, respectively. We decided to use an FA of 110° since the images acquired with a FA of 130° didn’t lead to significant visual improvement (Fig. 2).

**Fig. 3:**
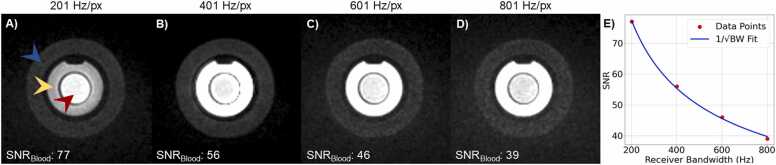
Phantom images of the receiver bandwidth test in a phantom. A), B), C) and D) show an axial slice at the isocenter for receiver bandwidths of 201, 401, 601 and 801 Hz/px, respectively. In A), The blue, yellow and red arrows point to compartments mimicking the tissue properties of myocardium, fat and blood respectively. E) SNR as a function of receiver bandwidth. Red dots indicate measured SNR values at various bandwidths, while the blue curve represents a square root fit, illustrating the expected inverse square root dependence of receiver bandwidth on SNR. While a receiver bandwidth of 201 Hz/px grants the highest SNR amongst all bandwidths tested, it led to significant banding artifacts and resultant loss of contrast in the images. *SNR* signal-to-noise ratio

The SNR measured on the phantom scans was 77, 56, 46 and 39 for receiver bandwidths of 201, 401, 601, 801 Hz/px, respectively. The receiver bandwidth also changed the TE/TR to 4.18/8.33, 3.08/5.94, 3.84/5.64, and 2.83/5.62 ms for receiver bandwidths of 201, 401, 601, and 801 Hz/px, respectively. Note that beyond a receiver bandwidth of 600 Hz/px, the prephasing gradient increase becomes longer than the time saved by shortening the readout gradient, so the TR increases. The image with a receiver bandwidth of 201 led to significant banding artifacts that decreased the contrast significantly and the receiver bandwidth that visually granted highest sharpness while having no noticeable banding artifacts on the images was 401 Hz/px (Fig. 3).

**Fig. 4:**
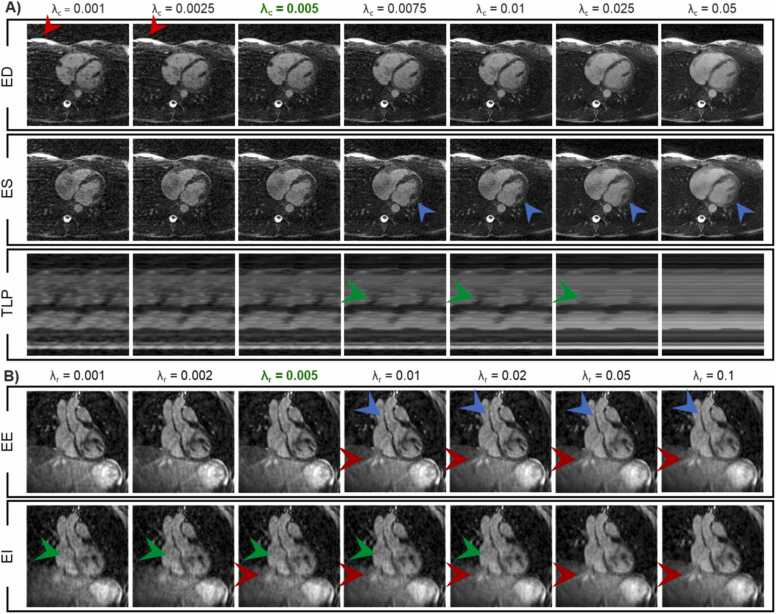
Reconstruction weight analysis, A) cardiac time dimension regularization weights, λ_c_, images in end systole ES and, ED and TLP and B) respiratory time dimension regularization weights, λ_r_, images in EE and EI. 5D CMR images were reconstructed using different weights ranging from 0.001 to 0.05. In A), The blue arrow points to part of the myocardium where motion blur can be observed when λ_c_ is high. The red arrow highlights the streaking artifacts originating from the chest fatty tissue and the green arrow shows temporal smoothing when λ_c_ increases. In B), the blue arrow points to the aortic vessel wall, which becomes blurry when λ_r_ is high. The red arrow points to a vein in the liver which appears in both end expiration EE and end inspiration EI meaning that the reconstruction failed to resolve respiratory motion when λ_r_ is too high. The green arrow points to the RV where higher regularization leads to higher SNR. *ES* end systolic, *ED* end diastole, *TLP* temporal line plot, *EE* end expiration, *EI* end inspiration, *5D* five-dimensional, *CMR* cardiovascular magnetic resonance, *RV* right-ventricular, *SNR* signal-to-noise ratio

The reconstruction parameters were tuned to reach the lowest amount of streaking artifacts without compressing the motion, which could lead to overestimating peak systolic volumes (Video S1). The cardiac and respiratory dimension total variation weights that visually granted the best results were 0.005 for both (Fig. 4).

Supplementary material related to this article can be found online at doi:10.1016/j.jocmr.2025.101906.

The following is the Supplementary material related to this article Video S1.

Video S1

### 3.2. Image quality

**Fig. 5:**
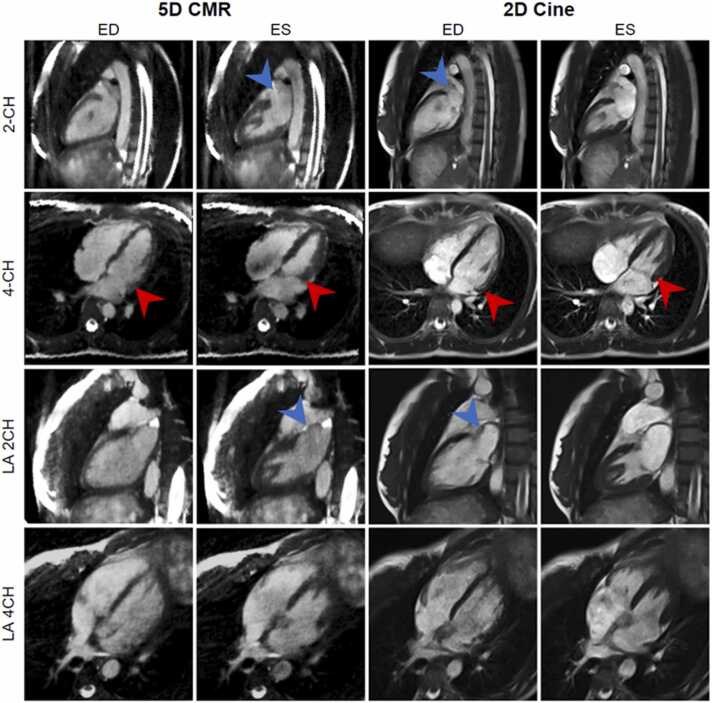
A comparison of ED and ES frames of 5D CMR and 2D cine in volunteer 1. LV wall motion on the ES and ED frames display a similar range of motion with both techniques which provides evidence that the heart motion was not compressed during reconstruction (red arrow). The LA-focused 2-CH and 4-CH views display larger volumes in systole and sharper LA wall when compared to the standard 2-CH and 4-CH view (blue arrow). *ED* end diastole, *ES* end systolic, *5D* five-dimensional, *CMR* cardiovascular magnetic resonance, *2D* two-dimensional, *LV* left ventricle, *LA* left atrial

**Fig. 6:**
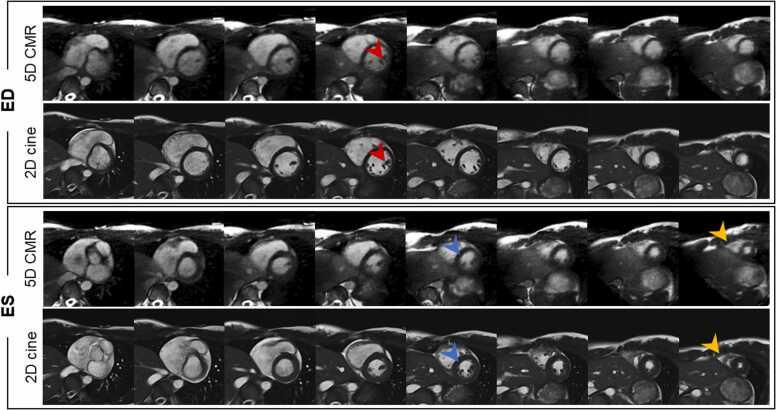
Short-axis stack slices in peak systole and end diastole, comparison between the 2D cine and 5D CMR acquisitions without contrast agent. 5D CMR SAX reformat resulted in blurrier blood-myocardium interface (blue arrow) but fine structures such as the in the papillary muscles were still resolved (red arrow). The yellow arrow points to the RV at the apex of the heart where the epicardium can be confused with RV blood pool in some cases for 5D CMR. *2D* two-dimensional, *5D* five-dimensional, *CMR* cardiovascular magnetic resonance, *RV* right ventricle

**Fig. 7:**
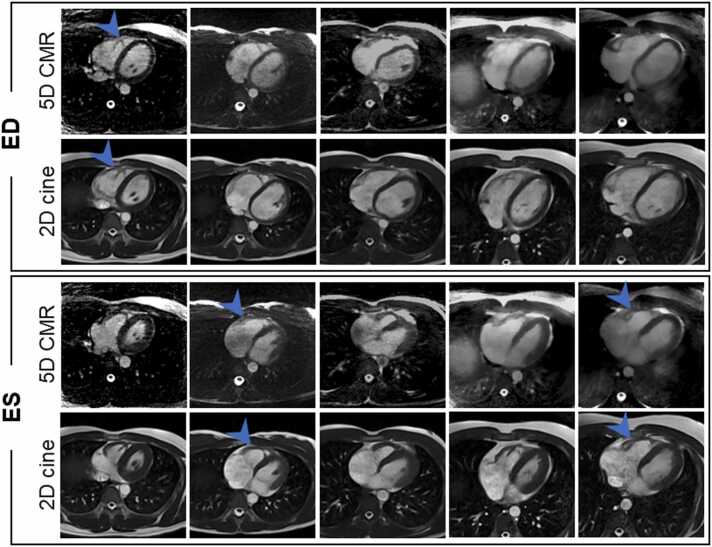
Axial slices comparing 5D CMR and 2D cines on five volunteers. 5D CMR axial slices have blurrier RV wall when compared to the 2D cines but, in both methods, myocardium motion and structural details in the papillary muscle are resolved (blue arrow). *2D* two-dimensional, *5D* five-dimensional, *CMR* cardiovascular magnetic resonance, *RV* right ventricle

The segmentation of the blood pool was feasible in all cases, although the reformatted 5D CMR images demonstrated lower visual image sharpness and contrast when compared to the 2D reference standard, (Fig. 5, Fig. 6 and Fig. 7). It took 60 ± 37 min to acquire all breath-held 2D cine slices, while the 5D CMR scan time was always 7 min 50 s. For some volunteers, the 2D cine acquisition time was lengthened because electrode placement needed to be adjusted, ECG filtering options were modified, or the ECG device was changed to another model. The need for multiple breath-holds, which ranged from 30 to 35 for the 2D cine, was eliminated in the case of the 5D CMR acquisition.

**Fig. 8:**
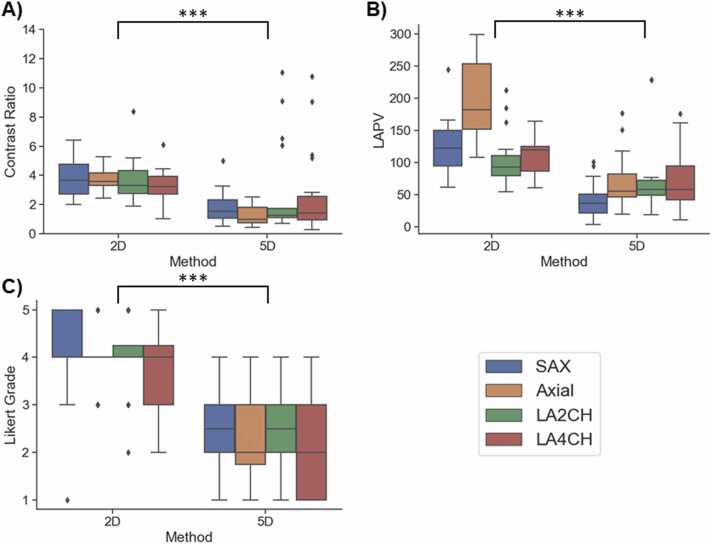
Contrast ratio (A), LAPV (B) and Likert score (C) boxplots for each orientation. No differences were found between orientations in all the metrics. Statistical significance computed with Wilcoxon signed rank test is indicated with the brackets, *** for p<0.001. The CR, LAPV and Likert grades measured on the 2D images were higher than that of the 5D images, p<0.05 for all. *LAPV* Laplacian Variance, *2D* two-dimensional, *5D* five-dimensional

The contrast ratio was lower on 5D CMR images (3.3 ± 2.9) than on 2D cines (4.7 ± 1.2) for every orientation, (p<0.05). However, the 5D CMR had a mean CR above 2 in all orientations (Fig. 8). The LAPV was 63 ± 60 in the 5D images compared to 135 ± 47 in the 2D cines (p<0.05, Fig. 8), which indicates lower sharpness for the 5D CMR images. The Likert grades amounted to 1.8 ± 0.8 for 5D CMR and 3.6 ± 0.9 for the 2D cines with grades of the 2D cine being significantly higher (p < 0.05, Fig. 8). The Likert grades appeared to degrade with higher BMIs and longer RR intervals, which also affects the signal intensity received by the coils and the undersampling factor, respectively (Fig. S1).

### 3.3. Volumes and ejection fraction

**Fig. 9:**
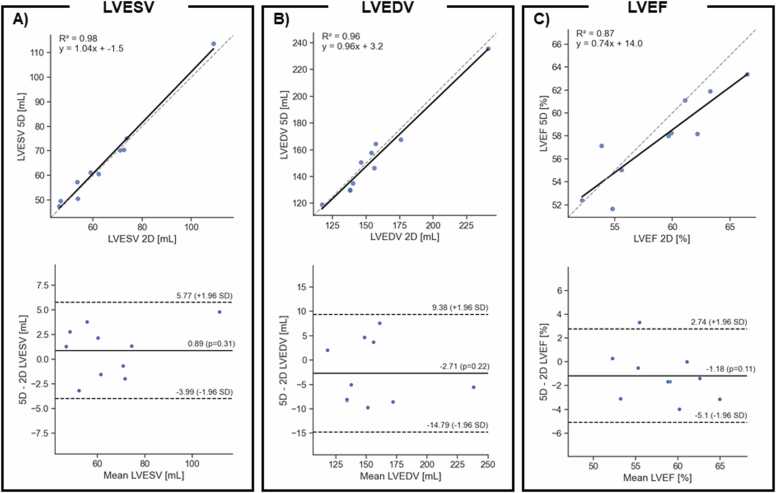
Correlation plot (top row) and Bland–Altman analysis (bottom row) of A) LVESV measured on the SAX cines, B) LVEDV measured on the SAX cines and C) LVEF obtained with the volume measurements. On the correlation plot the dashed line shows the identity line while the solid line shows the linear correlation. On the Bland–Altman plot; the dashed lines show the LoAs while the solid line represents the bias. *LVESV* left ventricular end-systolic volume, *SAX* short-axis stack, LVEF left-ventricular ejection fraction

The LVESV measurements means were similar with no statistically significant differences (67 ± 18 mL for 5D CMR vs 65 ± 18 mL for 2D cine, p = 0.38). The LVESV measurements had a strong correlation between the two methods, R^2^ = 0.98, while the LoAs were small, −4.0 to 5.8 mL (Fig. 9A). The LVEDV measurements means were similar with no statistically significant differences (153 ± 29 mL for 5D CMR vs 156 ± 32 mL for 2D cine, p = 0.16). The LVEDV measurements had a strong correlation between the two methods, R^2^ = 0.96, while the LoAs were small, −14.8 to 9.4 mL (Fig. 9B). Consequently, the LVEF measurements mean was in good concordance (58 ± 5% and 59 ± 5%, p = 0.49, Fig. 9). The correlation between the measured LVEF in 5D CMR and 2D cine images was strong (R^2^ = 0.87). The 95% LoA of the LV measurements ranged from –5.1% to 2.7% and the bias was −1.2% between the two methods (Fig. 9C).

On the SAX images, the RVESV measurement means were similar with no statistically significant differences (69 ± 15 mL for 5D CMR vs 67 ± 17 mL for 2D cine, p = 0.27). The RVESV measurements had a strong correlation between the two methods, R^2^ = 0.93, however, the LoAs were large, −29.4 to 18.0 mL (Fig. S2A). The RVEDV measurement means were similar with no statistically significant differences (151 ± 25 mL for 5D CMR vs 157 ± 32 mL for 2D cine). The RVEDV measurements had a strong correlation between the two methods, R^2^ = 0.93, while the LoAs were small, −7.8 to 11.7 mL (Fig. S2B). However, RVEF measurements means were significantly different when measured on the SAX images (56 ± 4% for 5D CMR and 58 ± 5% for 2D cine, p = 0.002), The SAX RVEF measurements were strongly correlated between the two methods (R^2^ = 0.91). On the SAX, the LoA for the RVEF ranged from –5.68% to 0.0% and the bias was 2.9% (Fig. S2 C).

**Fig. 10:**
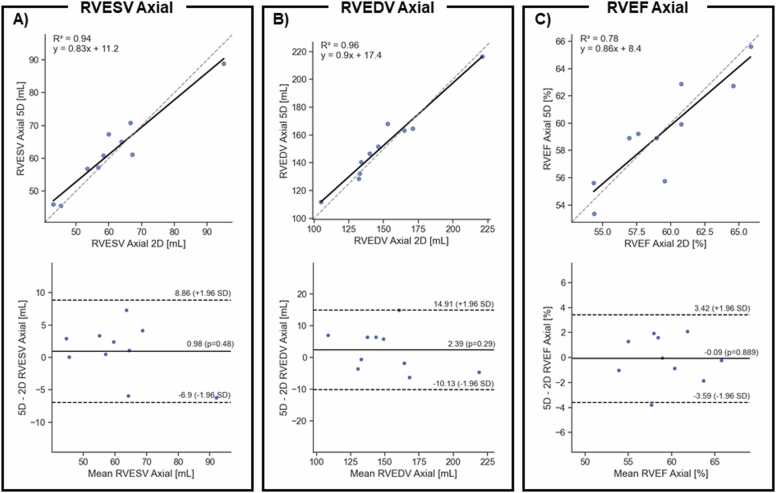
Correlation plot (top row) and Bland–Altman analysis (bottom row) of A) RVESV measured on the axial cines, B) RVEDV measured on the axial cines, C) RVEF obtained with the Axial volume measurements, D) RVESV measured on the SAX cines, E) RVEDV measured on the SAX cines, F) RVEF obtained with the SAX volume measurements. On the correlation plot the dashed line shows the identity line while the solid line shows the linear regression. On the Bland–Altman plot, the dashed lines show the LoAs while the solid line represents the bias. *RVESV* right ventricular end-systolic volume, RVEDV right ventricular end-diastolic volume, RVEF right-ventricular ejection fraction, *SAX* short-axis stack

On the axial views, the RVESV measurements means were similar with no statistically significant differences (63 ± 11 mL for 5D CMR vs 61 ± 14 mL for 2D cine, p = 0.50). The RVESV measurements had a strong correlation between the two methods, R^2^ = 0.94, however, the LoAs were small, −6.9 to 8.9 mL (Fig. 10A). The RVEDV measurements means were similar with no statistically significant differences (158 ± 26 mL for 5D CMR vs 150 ± 30 mL for 2D cine, p = 0.33). The RVEDV measurements had a strong correlation between the two methods, R^2^ = 0.96, while the LoAs were small, −10.1 to 14.9 mL (Fig. 10B). Consequently, the RVEF measurements means were in good agreement (60 ± 3% for 5D CMR and 60 ± 4% for 2D cine, p = 0.85). Similarly, the axial RVEF measurements were also correlated (R^2^ = 0.78) while the LoA for the RVEF ranged from –3.6% to 3.4% and the bias was −0.1% (Fig. 10C).

**Fig. 11:**
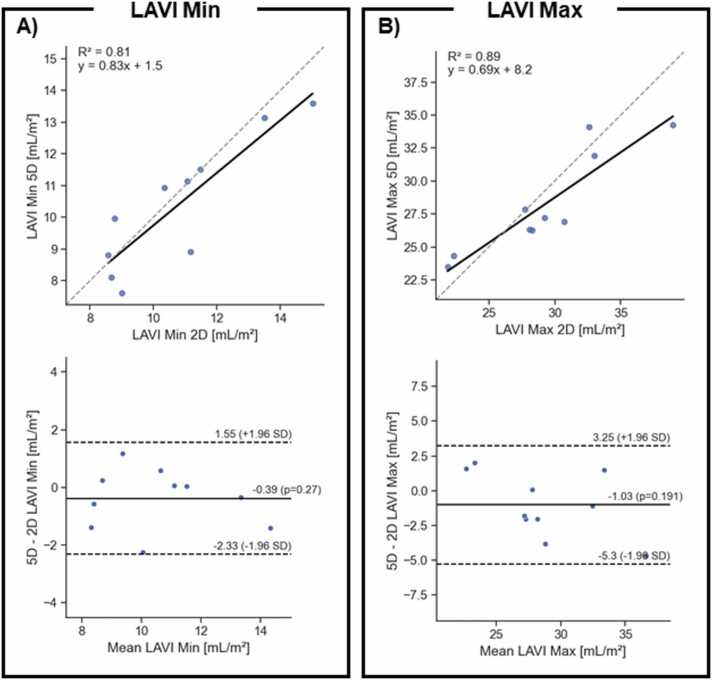
Correlation plot (top row) and Bland–Altman analysis (bottom row) of A) LAVI min measured on the dedicated LA2CH and LA4CH views, B) LAVI max measured on the dedicated LA2CH and LA4CH views. On the correlation plot, the dashed line shows the identity line while the solid line shows the linear regression. On the Bland–Altman plot, the dashed lines show the LoAs while the solid line represents the bias. *LAVI* left atrial volume index

The LAVI_min_ measurement mean was 10.6 ± 2.4 mL/m^2^ when measured on the 5D CMR images and 10.8 ± 2.2 mL/m^2^ on the 2D cines (p = 0.9). The LAVI_min_ measurements were also correlated (R^2^ = 0.81) while the LoA ranged from –2.3 to 1.6 mL/m^2^ and the bias was −0.4 mL/m^2^ (Fig. 10A). The LAVI_max_ volumes were similarly in good agreement with 29.2 ± 3.9 mL/m^2^ on 5D CMR images and 29.3 ± 5.0 mL/m^2^ on the 2D cines (p = 0.6). Similarly, the LAVI_max_ measurements were also correlated (R^2^ = 0.89) while the LoA for the LAVI_max_ ranged from –5.3 mL/m^2^ to 3.25 mL/m^2^ and the bias was −1 mL/m^2^ (Fig. 11B).

### 3.4. Qualitative assessment

The 5D CMR images showed similar structural details in the papillary muscles when compared to the 2D cines (Fig. 3). However, the mitral valve was blurred and unnoticeable on those images. The atrial wall was sharper and had more contrast in the LA-focused views for both methods (Fig. 3, blue arrow). On the SAX cine comparison (Video S1), the motion range was identical for both methods. Slight streaking can be observed on the edges of the 5D CMR images and flow artifacts were present during the early ventricular diastole. The RV wall had excellent contrast and sharpness with both methods on the axial slices (Fig. 6, blue arrow). The observed range of motion and structural details were similar with both methods. The RV wall was also easier to segment on axial views especially towards the apex of the heart, where the RV can be confused with epicardium on the SAX (Fig. 5). Respiratory motion videos were extracted for all volunteers and end expiration was found to be the best reparatory phase for the measurement of Biventricular and LA volumes (Video S2).

Supplementary material related to this article can be found online at doi:10.1016/j.jocmr.2025.101906.

The following is the Supplementary material related to this article Video S2..

Video S2

## 4. Discussion

We demonstrated that 5D CMR supports the measurement of cardiac function on a 0.55T clinical system with moderate gradient performance. 5D CMR provides quantitative LV, RV, and LA functional analysis results that are consistent with those from the 2D reference standard, while the 5D data were collected in under 7 min 50 s using a single volume placement and mouse-click to initiate the scans.

The low-field system used in this study offers the advantage of an 80 cm wide bore, which may be beneficial for obese or claustrophobic patients. Additionally, the lower main magnetic field, along with moderate dB/dt, helps to reduce acoustic noise during the scan, which further supports improved patient comfort [25], [26]. 5D CMR is more time-efficient, eliminates the need for ECG placement, breath-holding, navigators, or respiratory bellows and therefore significantly reduces operator and patient involvement, and abbreviates the scan duration decisively. This advances the hypothesis that 5D CMR will be less operator dependent and that there will no longer be a requirement for highly trained experts to collect CMR data which in turn could make CMR exam more cost competitive when compared to other techniques such as Computed Tomography (CT) or echocardiography.

5D CMR also provides an isotropic 3D volume that can retrospectively be reformatted into any anatomical views, thus removing the need for complex slice planning during the exam. This is also advantageous for the accuracy of LA volume measurements, which are more precise with the LA-focused views, and the RVEF, which is more precise on axial stack of cine. Both axial stack and LA focused views are not performed routinely in CMR and would lengthen the exam. Reformatting the slices off-line pushes the slice planning from the scanner to a standard computer. It reduces scan time which might increase the patient throughput and therefore the cost-effectiveness of CMR exams. The segmentation of the myocardium and blood pool were performed in 8mm-thick 2D slices which does not exploit the full potential of the 3D volumes acquired with the 5D CMR method. Using thinner slices or 3D segmentation techniques, more accurate measurements may be obtained by taking advantage of the higher isotropic resolution and the isotropic 3D volume [27]. Especially, an automated deep learning segmentation could extend the one-click imaging pipeline to a one-click heart function analysis. Another limitation of this study is the lack of comparison between 5D CMR at 0.55T and the clinical standard cine at higher field strengths. Here, we compared the 5D CMR with 2D cine at 0.55T, however, it has already been shown that cines acquired on 0.55T scanners were on par with those acquired on 1.5T systems for the assessment of LVEF and RVEF [28], [29].

This study provides promising preliminary results for low field 5D CMR which warrants additional studies in patient cohorts where specific conditions can be analyzed and compared between the two methods.

## 5. Limitations

The images acquired with the proposed technique had lower image quality, as quantified with both the contrast and the sharpness. Both can be attributed to the reduced inflow contrast and the higher undersampling factor at the periphery of k-space using 5D CMR. We noticed a trend of quality degrading with high BMI and long RR intervals which might need further investigation in a patient population suffering from cardiovascular diseases. Instead of the fixed 50 ms cardiac bin duration, using a predetermined number of cardiac temporal bins would mitigate the effects of RR variability on image quality and improve the robustness of the technique.

Further optimization of the sequence parameters, different k-space trajectories, such as spirals, which benefits from the prolonged T2* at 0.55T [30], and more advanced reconstruction techniques [31] might compensate for those factors and bring 5D CMR image quality and contrast closer to those of the 2D cines. More specifically, recent advances in deep learning reconstructions might offer potential for higher undersampling rates and higher image quality simultaneously [32].

The volumes were measured on interpolated 2D slices which may induce distortion. The images remained visually very similar, especially in the center of the FOV and around the heart. Distortions, such as pixelation and stretching, was only observed at the periphery of the FOV and mostly in orientations along the z axis, e.g. in the 2CH view. Using 3D segmentation techniques would avoid distortion issues and take advantage of the full 3D cine. Some distortions may result from gradient nonlinearities, which could be corrected using pre-measured distortion maps [33]. Post hoc gradient correction algorithms, such as GIRF can mitigate gradient-induced trajectory errors and associated image artifacts, potentially improving overall image quality [34], [35], [36]. The LAVI data showed fairly strong correlation between the two methods, however, the LoA are relatively large which might be a limiting factor for accurate LA assessment. Using thinner slices, 3D segmentation or denoising techniques might improve the results and lower the LoA.

The reference 2D exam comprised both SAX and Axial stacks which significantly increased the number of acquired slices and therefore the total duration when compared to a routine CMR exam. Furthermore, the reference method in this study may not encompass the full range of protocols used for 2D cine acquisition in routine biventricular assessment. Alternative techniques, such as free-breathing 2D cines with multiple averages, may, like 5D CMR, enhance patient comfort while ensuring accurate evaluation. However, these protocols are still emerging on low-field scanners with limited gradient performance. Additionally, a more time-efficient 2D cine pulse sequence, fully integrated ECG devices optimized for workflow, and technicians trained to acquire the LA2CH and LA4CH views could contribute to shortening the examination duration and reduce the difference between 2D and 5D examination time. Nevertheless, the 5D approach offers the advantage of whole-organ data collection during free breathing with a fixed scanning time, eliminating the need for ECG electrode placements and pauses between breath holds. As a result, acquisition time and its variability are significantly reduced while workflow is improved.

While we only extracted the end-expiratory images, studying ventricular function at different respiratory levels may be investigated as a new biomarker [37], [38], [39]. Simultaneously, algorithms such as iMoCo [40] or fNAV [41] may support the combination of data originating from different respiratory levels in the interest of SNR improvement.

## 6. Conclusion

This study demonstrates the feasibility of accurately measuring LV, RV, and LA Volumes in a 7:50 min single-click 5D CMR scan on a low-field 0.55T with limited gradient performance. Despite lower image quality compared to conventional breath-held 2D cine, similar quantitative results were demonstrated. 5D CMR can thus greatly simplify acquisition by obviating the needs for breath-holding, ECG placement and slice-planning.

## Funding

This study was funded by the 10.13039/501100001711Swiss National Science Foundation (grant numbers 320030–227737 [to M.S.]; 206021_205413 [to M.S.]; 320030B_201292 [to M.S.]; 201292 [to M.S.]; 202140 [to C.W.R.]; and 182615 [to R.B.v.H.]) and the National Institutes of Health (grant number R01HL161618 [to O.P.S.]). O.P.S. is supported by the John W. Wolfe and Edgar T. Wolfe Foundation.

## Author contributions

**Xavier Sieber:** Writing – original draft, Visualization, Validation, Software, Methodology, Investigation, Formal analysis, Data curation. **Katherine Binzel:** Investigation, Data curation. **Juliet Varghese: Supervision, Methodology, Investigation. Yingmin Liu:** Supervision, Investigation. **Jerome Yerly:** Writing – review & editing, Software. **Christopher W. Roy:** Writing – review & editing, Software. **Panagiotis Antiochos:** Writing – review & editing, Data curation. **Milan Prsa:** Writing – review & editing, Data curation. **Ruud B. van Heeswijk:** Writing – review & editing, Supervision, Project administration, Methodology. **Orlando P. Simonetti:** Writing – review & editing, Supervision, Resources, Project administration, Methodology, Funding acquisition. **Matthias Stuber:** Writing – review & editing, Supervision, Project administration, Methodology, Funding acquisition.

## Declaration of competing interests

The authors declare that they have no known competing financial interests or personal relationships that could have appeared to influence the work reported in this paper.
