# Supplementary material for: Measuring biventricular function and left atrial volume in a single five-dimensional whole-heart cardiovascular magnetic resonance scan at 0.55T
Source: J Cardiovasc Magn Reson. 2025 May 8;27(1):101906. doi: 10.1016/j.jocmr.2025.101906 (PMC12166699; doi:10.1016/j.jocmr.2025.101906)
Supplement: Supplementary file 3 — Supplementary material [file mmc1.docx]

# Supplementary Material

**Video S1:** Example of SAX cine on one volunteer acquired with both methods. Both methods show a similar range of motion and good blood-myocardium contrast. The 5D CMR images suffer from more artifacts originating from the bright fat signal of the chest.

**Video S2:** 5D CMR respiratory video examples on volunteer 1, 4, 6 and 9. The respiratory frame in end expiration has the lowest level of streaking artifact and higher sharpness. Therefore, it was selected for the quantitative analysis in all volunteers.


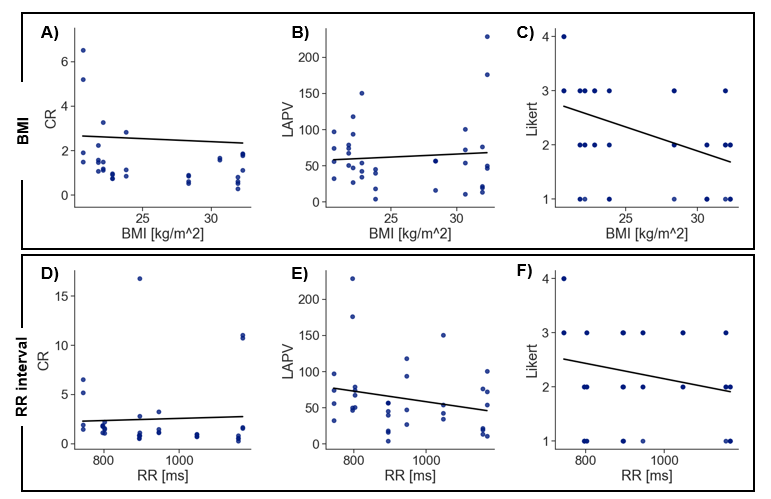


**Figure S1:** Quantitative image quality parameters (A and D: CR, B and E: LAPV, C and F: Likert score) as a function of patient specific physiological parameters (BMI and RR interval). Likert score and end diastolic images. The blue dots represent each data point while the black line is a linear fit of the data.


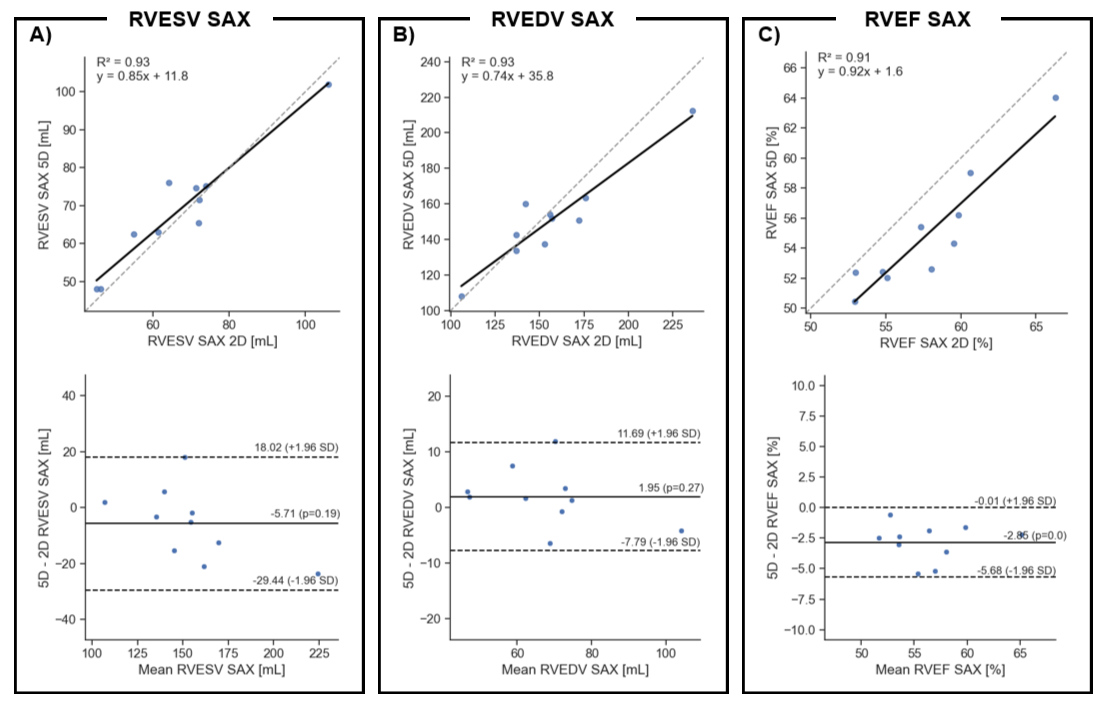


**Figure S2:** Correlation plot (top row) and Bland-Altman analysis (bottom row) of A) RVESV measured on the SAX cines, B) RVEDV measured on the SAX cines, C) RVEF obtained with the volume measurements. On the correlation plot the dashed line shows the identity line while the solid line shows the linear regression. On the Bland-Altman plot, the dashed lines show the LoAs while the solid line represents the bias.
